# Rapid brain structure and tumour margin detection on whole frozen tissue sections by fast multiphotometric mid-infrared scanning

**DOI:** 10.1038/s41598-021-90777-4

**Published:** 2021-05-28

**Authors:** Tim Kümmel, Björn van Marwick, Miriam Rittel, Carina Ramallo Guevara, Felix Wühler, Tobias Teumer, Björn Wängler, Carsten Hopf, Matthias Rädle

**Affiliations:** 1 Center for Mass Spectrometry and Optical Spectroscopy (CeMOS), Mannheim University of Applied Science, Paul Wittsack-Str. 10, 68163 Mannheim, Germany; 2 Molecular Imaging and Radiochemistry, Department of Clinical Radiology and Nuclear Medicine, Medical Faculty, Mannheim of Heidelberg University, Theodor-Kutzer-Ufer 1-3, 68167 Mannheim, Germany

**Keywords:** Imaging, Optical spectroscopy, Structure determination, Cancer imaging, Lipids, Proteins, Cancer, Medical imaging, Optics and photonics, Applied optics, Lasers, LEDs and light sources, Optical techniques, Biomedical engineering, Scientific data, Applied physics, Optical physics, Techniques and instrumentation, Surgical oncology

## Abstract

Frozen section analysis is a frequently used method for examination of tissue samples, especially for tumour detection. In the majority of cases, the aim is to identify characteristic tissue morphologies or tumour margins. Depending on the type of tissue, a high number of misdiagnoses are associated with this process. In this work, a fast spectroscopic measurement device and workflow was developed that significantly improves the speed of whole frozen tissue section analyses and provides sufficient information to visualize tissue structures and tumour margins, dependent on their lipid and protein molecular vibrations. That optical and non-destructive method is based on selected wavenumbers in the mid-infrared (MIR) range. We present a measuring system that substantially outperforms a commercially available Fourier Transform Infrared (FT-IR) Imaging system, since it enables acquisition of reduced spectral information at a scan field of 1 cm^2^ in 3 s, with a spatial resolution of 20 µm. This allows fast visualization of segmented structure areas with little computational effort. For the first time, this multiphotometric MIR system is applied to biomedical tissue sections. We are referencing our novel MIR scanner on cryopreserved murine sagittal and coronal brain sections, especially focusing on the hippocampus, and show its usability for rapid identification of primary hepatocellular carcinoma (HCC) in mouse liver.

## Introduction

The significance of rapid analysis of tissue sections and the detection of defined morphological structures, benign and malignant regions as well as tumour margins in frozen sections is increasing^1^. Simultaneously, the reliability of the rapid analysis on frozen tissue sections should be reproducible and the measurement results distinct^2,3^. This common challenge is not limited to laboratories, it is prevalent in clinical environments such as the rapid intraoperative pathologic examination of tissue sections during surgery^1–4^. Laboratory molecular examinations of tissue sections, performed apart from clinical applications, usually require the evaluation and pre-analysis of sections to be examined as a helpful or basic requirement^5^. In general, this relates to the use of procedural complex measurement processes for molecular analysis, such as mass spectrometry imaging^6^. Extended measurement processes can be significantly reduced by limiting the region of interest (ROI), resulting in less data load and reduced calculation time. Thus, pre-processing tissue and data is recommended prior to the main sample analysis. Even though pre-analysis provides reduced information, it is sufficient to limit the ROI^6–8^. There are well-established pre-analysis methods such as FT-IR imaging^6^, various confocal microscope setups^9–12^ or Raman-based measuring techniques^13,14^. These are usually label-free and aim for low data loads in combination with reduced measurement times at maximum information content. The focus is often on selected wavelengths that are customised for the respective application. Currently, these methods are used for rapid analysis of tissue sections. However, these imaging procedures require significant processing time associated with a reduced measuring range on the sample (< 1 cm^2^) and high spatial scanning resolution. Measurement setups based on microscope technology currently provide a minimized measurement time and are usually designed for transmitted and confocal reflected light examinations^12,15,16^. Reflection-based microscopic systems require hematoxylin and eosin (H&E) staining or various fluorophores to achieve optimal molecule dependent analysis results^17^. Furthermore, the related sample preparation is work intensive. Raman-based tissue scanners exhibit high detection sensitivity and are well suited for tissue section analysis^18^. However, low measurement times can only be reached for small (scanning) areas. Therefore, they are not competitive with established scanning methods, that are suitable to analyse whole tissue sections with a pathological assessment in efficient time^18–20^.

In clinical settings, rapid tissue analysis is also required during surgery. Especially in case of tumour resection, such as skin cancer^21^, lymph nodes^22,23^ or liver tumours^24–26^, pathological examination is performed during surgery to determine the progress of tumour resection. In general, the analysis of the sample is performed by H&E staining and has to be evaluated by a pathologist. This evaluation of the degree of tumour removal can be made intraoperatively^27^. The aim is to extirpate the preoperatively diagnosed findings while reliable intraoperative assessment of a frozen section^1^. Although this method is widely used to analyse frozen sections, the analysis is time-consuming and interrupts the running surgical process^22^. Furthermore this method is the current approved methodology, the pathological evaluation of H&E stained sections is subject to inaccuracy^28–30^. Independent studies proved that tissue sections of the same type can be assessed subjectively by several pathologists in different ways and therefore can result in misdiagnosis. In certain cases, a tumour can be classified as malignant or benign or neither, leading to incorrect treatment of the patient. Intraoperative misdiagnosis of pathological tissue sections inevitably results in an unsuitable progress of the surgery^31–33^.

The aim is to find typical tissue structures or tumour margins by rapid chemical segmentation in order to select areas of interest for further more precise investigations. This is valid for both laboratory and clinical applications^1,6^. The tumour and its rough location is known in most cases by preoperative examinations. Anyway, the precise boundaries to adjacent known tissue are not entirely defined.

Therefore, a reliable measurement procedure that treats with fast measurement at large measuring range (> 1 cm^2^) and less data load is essential. In addition, the spatial resolution has to be as high as technically feasible. This is the balance in this project. In this work we show that we have succeeded in dealing with this problem and in developing a measuring method that perform these requirements. This is not about classifying tumours into tumour types or conducting in-depth molecular investigations. We focus on the rapid segmentation of general tissue structures. This includes the rapid recognition of tumour margins in tissue sections. We are validating and testing our measurement system on brain structures and thus demonstrate that the measurement method is competitive with the already established FT-IR imaging method. Subsequently, we present a scanning method that is capable of detecting naturally grown liver carcinoma.

## Results

Base for our experimental procedures is a novel MIR scanner, that will be integrated into a common workflow for frozen section analysis. In order to clarify the significance of the MIR scanner, its properties and functions are described in the following. An overview of the workflow integrates the developed scanning system into a familiar workflow of tissue analysis. The measurement results are focused specifically on the detection of the hippocampus in the coronal plane of mouse brain and the recognition of tumour margins in mouse liver.

### Middle infrared scanner

The optical measuring system presented here consists of a flying spot scanner^34–36^. As previously published, the system is already designed to scan a sample surface with a non-contact technique^37,38^. Here the measuring system is based on two laser wavenumbers that are feasible for detection of long-chained C-H molecules. The related experiments referred specifically to polymers and body fat of animals. To use the application presented in this work, the laser unit was extended by two to a total of four lasers. Thus, the scanning process is performed with four wavenumbers in the mid-infrared range.

**Figure 1:**
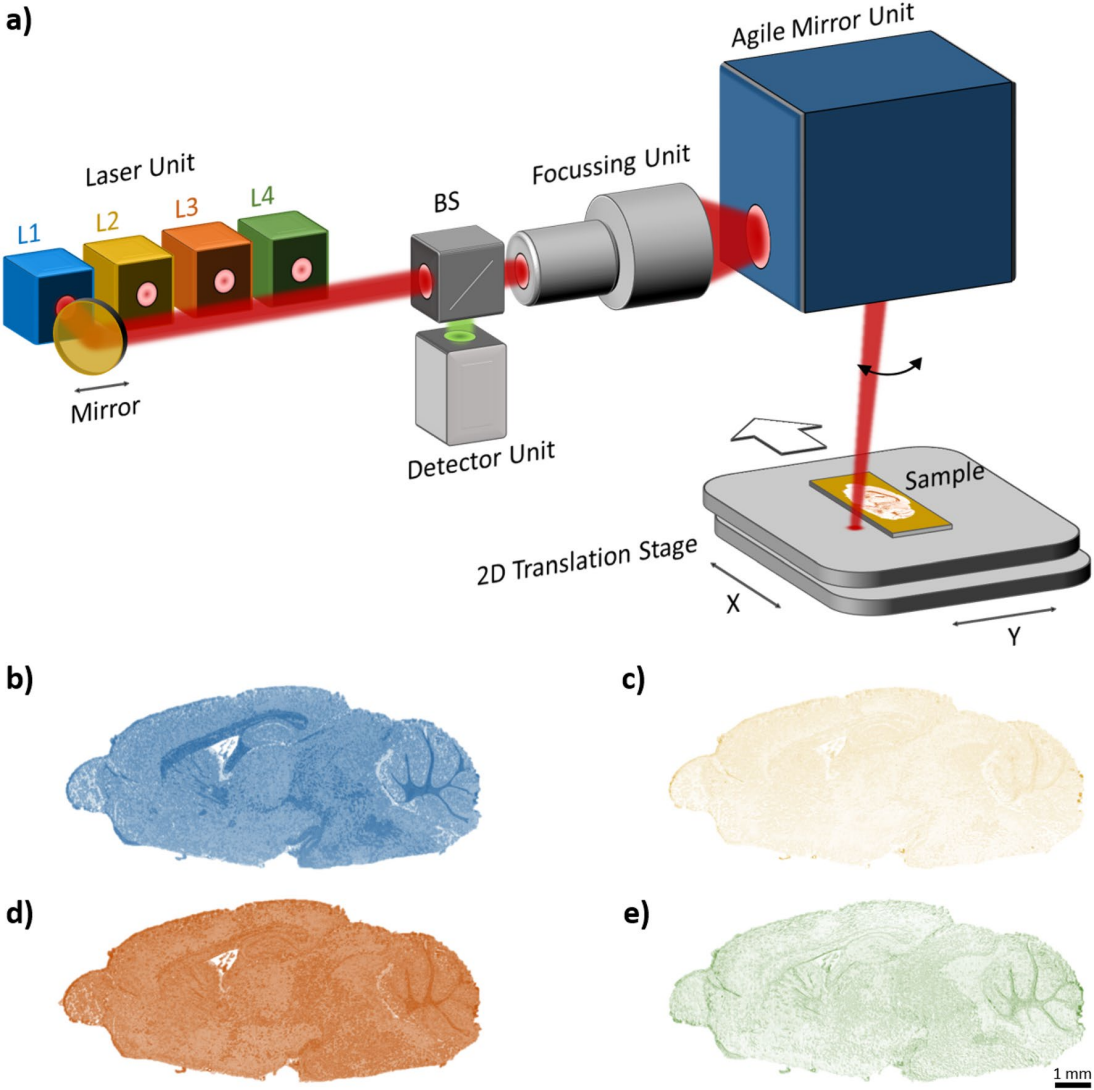
Schematic representation of the MIR scanner with corresponding measurement results of a sagittal sectioned mouse brain C57BL/6. (**a**) Experimental setup consisting of laser unit, focus unit, scan system with agile mirror system and IR-detector unit. (**b**) Scan result for target wavenumber 2926 cm^−1^, laser 1. (**c**) Scan result for reference wavenumber 2790 cm^−1^, laser 2. (**d**) Scan result for target wave number 3350 cm^−1^, laser 3. (**e**) Scan result for reference wavenumber 3700 cm^−1^, laser 4.

The wavenumbers (2790 cm^−1^/~ 3.6 µm, 2926 cm^−1^/~ 3.4 µm, 3350 cm^−1^/~ 3 µm, 3700 cm^−1^/~ 2.7 µm) of the four distributed feedback (DFB) lasers (nanoplus Nanosystems and Technologies GmbH, Germany) are selected to ensure that one laser wavenumber is absorbed by lipids (target laser lipids). The other laser emits a wavenumber that absorbs less to almost none lipids (reference laser lipids). A similar laser combination is applied to protein vibration bands. This results in two target lasers and two reference lasers. The interpretation of the correct wavenumber requires the observation of absorption bands of lipid and protein vibration in the spectral range. The spectral range in the infrared (IR), especially the mid-infrared, is classified into functional groups and the fingerprint region^39^. These two areas include the most important vibrations of molecules. The individual vibration types such as deformation vibration and valence vibration are located in the fingerprint region. In the functional group range, the valence vibrations for lipids are particularly important, since they have extremely strong vibrations in this range. Valence vibrations of lipids have a vibration peak at 2926 cm^−1^^40,41^. For proteins, the vibration band varies between 3200 cm^−1^ and 3500 cm^−1^ of the functional groups^41^, which depends on the type of protein. The selected laser wavenumbers for the MIR scanner setup (Fig. 1) are 2790 cm^−1^ (reference lipids, L1), 2926 cm^−1^ (C–H vibration bond for target lipids, L2), 3350 cm^−1^ (N–H vibration bond for target proteins/amide A, L3) and 3700 cm^−1^ (reference proteins, L4). Basically, the target lasers are sufficient for later clustering and segmentation of the scan results. In this case, the reference lasers represent sufficient scattering properties of the sample, whereas the target lasers contain the absorption properties of the sample. A combination of the reference and target lasers improves the subsequent clustering by provides more spectral information about the sample.

The lasers (L1 to L4) are used in sequence for the following measurements (Fig. 1a). A mirror is mounted on a slide on a linear axis moves automatically from one laser module to the next and thus couples the selected laser into the optical focus unit. The focused laser beam is guided on the sample by an agile mirror unit. A scanning field is achieved by orthogonal movement of the sample by means of a 2D translation stage. The measuring system acquires the confocal direct reflex via an infrared (IR) detector. The detection signal is transferred from the optical system via beam splitter (BS). This enables the setup to run at a sampling rate of ~ 2.7 MS/s (including oversampling) with a spatial resolution of 20 µm. The spatial resolution is determined by means of calibration targets with defined resolution patterns. The spatial resolution results from the mirror velocity of the agile mirror unit and the velocity from the translation stage. As a result of the mirror movement, the laser is deflected a defined and calibrated pathway on the sample. On this resulting laser line, a certain number of measuring points is acquired. The temporal acquisition of the individual measuring points results in the spatial resolution. To ensure a constant resolution, the data acquisition is triggered on each new laser line generated by the align mirror unit. In combination with the constant translation stage velocity, a measurement field with the specific spatial resolution is then generated. Hence, the scans are performed by scanning defined lines on the sample while the translation stage is moving orthogonally. This results in an array of pixels represented by a measurement image. The measuring time for scanning a sample area of 1 × 1 cm^2^ is at 3 s. The following measurements are based on a spatial resolution of 20 µm. The scanning time increases proportionally while the scanning range increases and the spatial scanning resolution remains constant. Compared with other proposed measurement systems, our measurement method is much faster and can be integrated into routine tissue testing. Moreover, this method is not limited to transparent samples for transmission light examination. The MIR scanner is primarily intended for the approximate detection of tumor margins on spectral data basis. Depending on the application and need, a high-resolution examination (spatial resolution less than 5 µm) can be performed using an additional measurement method, such as high-resolution infrared microscopy^16^. In this case, an additional measurement process can be performed, based on the measurement data of the MIR scanner and its preselected ROI.

The fundamental feasibility of the MIR scanner for tissue imaging is shown in Fig. 1b–e. These are scan results of brain from mouse type C57BL/6 in sagittal plane. Each scan result includes 400k measurement points, acquired within ~ 4.8 s. The measurement images are additionally coloured for this illustration to indicate the different laser wavenumbers. The measurement images show significant differences for each laser used. Target (Fig. 1b,d) and reference (Fig. 1c,e) wavenumbers are distinguished from each other based on the measurement data, since the intensity of the pixels represents the absorbance. The scan results are normalized and calculated with a previously acquired background measurement. Since these measurement results just represent a demonstration of the MIR scanner, the results are not discussed in detail in this case.

### Workflow

**Figure 2:**
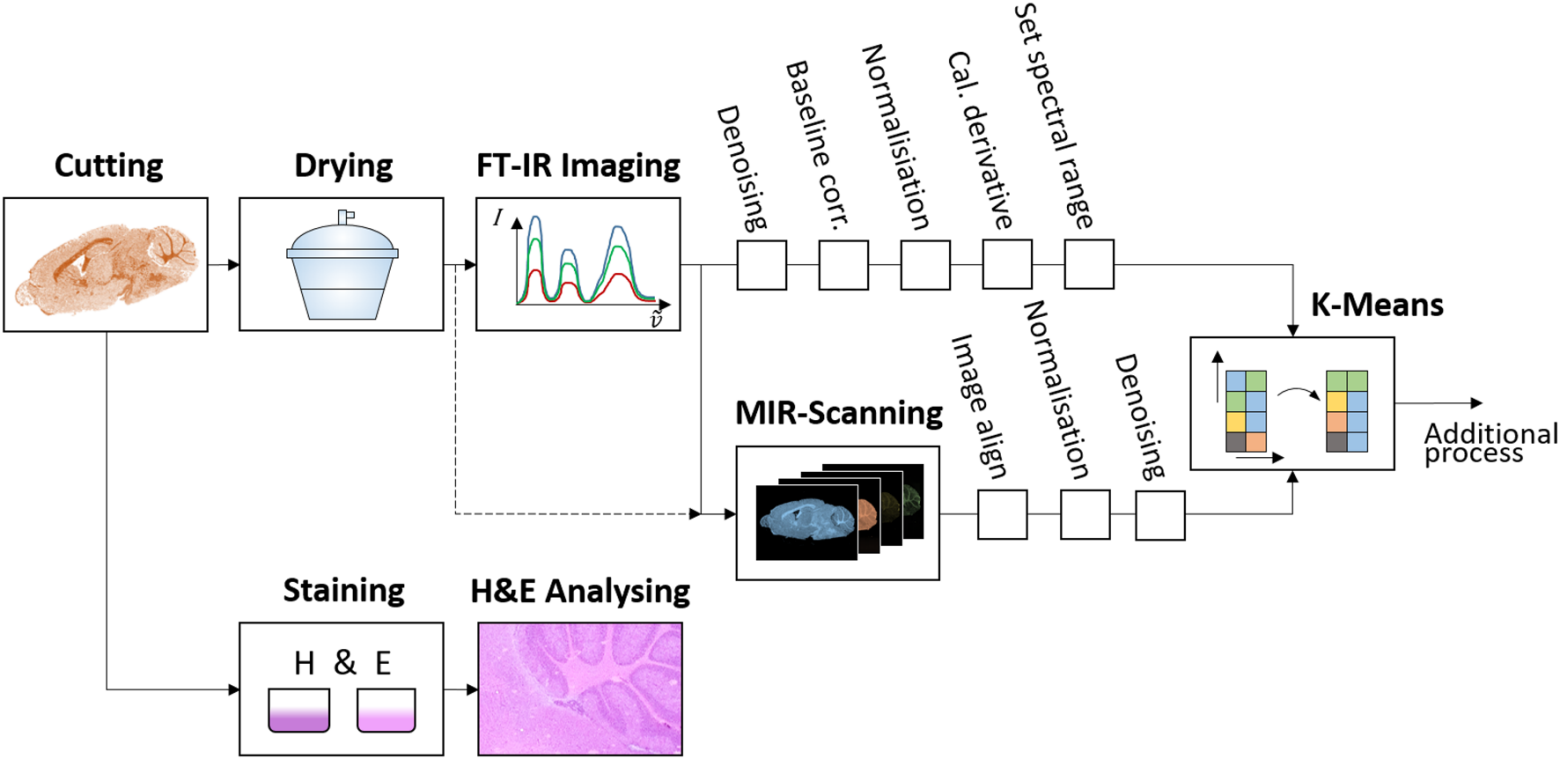
Schematic experimental workflow for precise comparison of two IR scanning systems. Two adjacent tissue sections are used. After drying one section was used for spectral measurements by FT-IR imaging followed by MIR scanning. After data pre-processing clustering by k-means was performed. The second tissue section was H&E-stained for structure referencing.

Two adjacent sections of the respective tissue are prepared for the investigations. One section is used for spectral measurement with FT-IR imaging followed by MIR scanning as shown in Fig. 2. The second section is used for H&E staining for better visibility of morphological structures of the respective tissue. Before the spectral measurement, drying is performed in a desiccator for 10 min. Drying is necessary to minimize the water content in the sample. Otherwise, the O–H vibration superposes the C–H and N–H valence vibration. Thus, a reliable measurement of the C–H and N–H bands in the functional group region is impossible. The subsequent FT-IR imaging acquires a full spectrum (4000 cm^−1^ until 750 cm^−1^ wavenumbers) and is used as a reference system. In comparison, the novel MIR scanner method is using only the previously described wavenumbers. A comparison is performed between established FT-IR imaging method^6^ and the novel MIR measuring method. Algorithms like denoising (locally adaptive total variation regularization^42^ applied to the measurement images) and normalisation prepare the mid-infrared data of both scanning methods for clustering. For each scanning method the processed measurement data are sorted by k-means algorithm^43^. The resulting clustering and segmentation provides related data that can be associated with interrelated structures. The advantage of this method is that multiple wavenumbers are viewed simultaneously and related data sets are displayed combined in a single image^6^. Thus structures of different wavenumbers become visible. Subsequently, further complementary processes can be added as shown in a previous work^6^.

Based on the H&E image the referencing of clustered FT-IR and MIR images is feasible. In addition, H&E sections are used to evaluate which structures are better visible with the respective scanning method. At the end of our experiments we assume that the MIR scanning can also substitute the FT-IR process, which is indicated by the dashed line in Fig. 2.

### Clustering the hippocampal region

**Figure 3:**
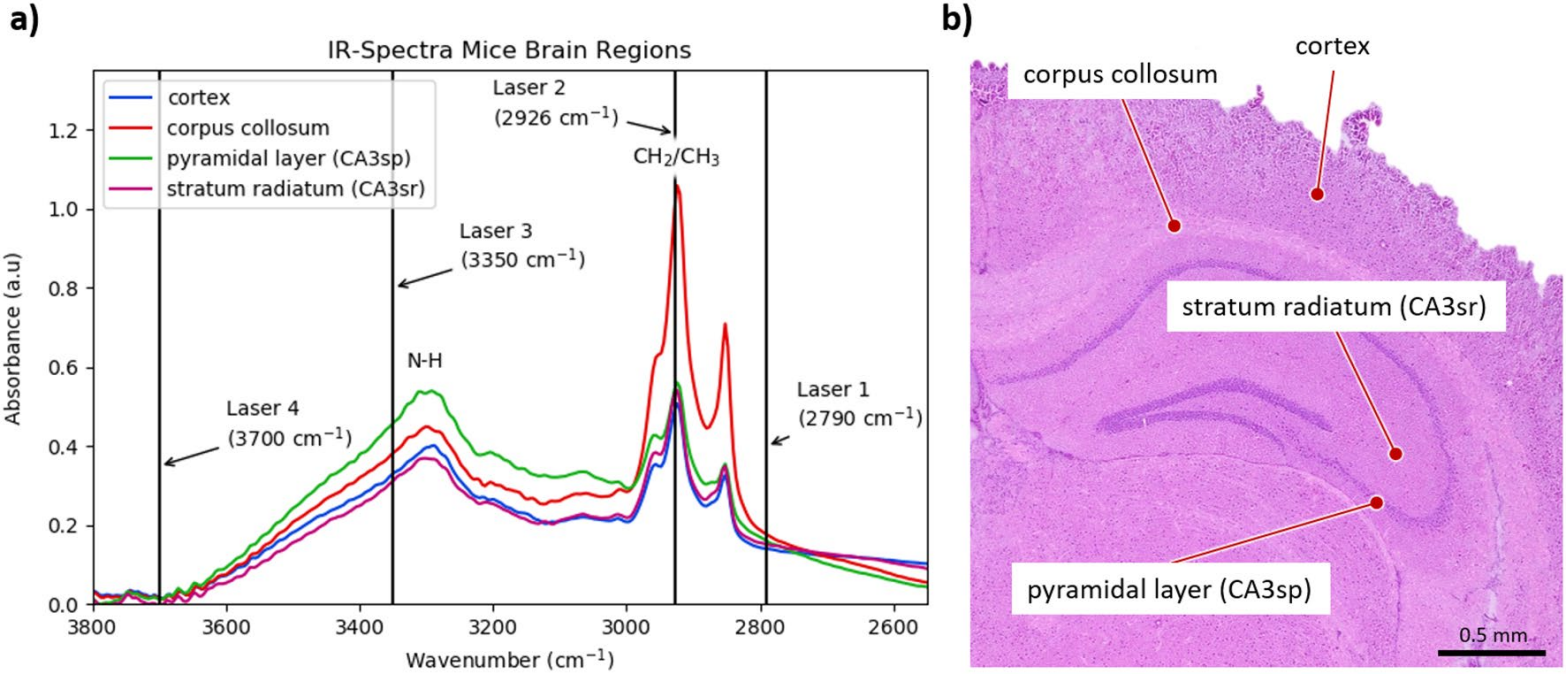
Reference measurement of characteristically mouse brain structures. (**a**) IR spectra of cortex and corpus collosum as well as the CA3 region of coronally sectioned brain from mouse type C57BL/6. These spectra are performed by FT-IR imaging and references the used laser combination. (**b**) Location of measurement points presented in H&E stained section.

For segmentation and clustering of the hippocampus, the coronal section of a brain from mouse type C57BL/6 is used. This experiment does not require all wavenumbers integrated in the MIR scanner. Figure 3a shows the spectral properties of selected parts of the mouse brain. The data are acquired by FT-IR and refer to measurement points within grey (cortex) and white (corpus collosum) matter^44^ as well as in the hippocampus (Fig. 3b), especially in the CA3 region^45^. In spectral observation, the spectral information of laser 1 and laser 4 does not differ except for an offset. It is not required to use both lasers in this case. Therefore, MIR measurement results are presented for three wavenumbers. Laser 2 and laser 3 are significantly different in absorbance. This indicates that protein and lipid content change for each measured substance. Though laser 3 is not located exactly on the vibrational peak of proteins (N–H), the absorption difference is still sufficient to distinguish the individual substances.

**Figure 4:**
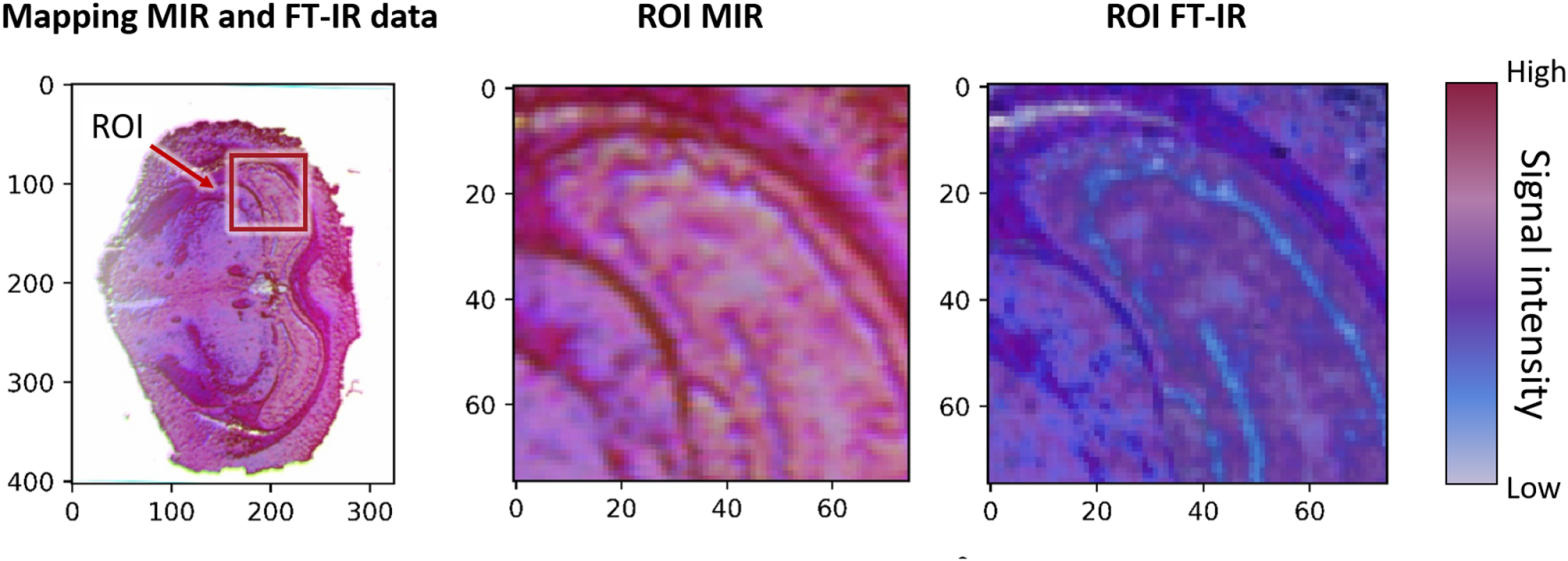
Computation of the similarity between MIR and FT-IR data. The compared wavenumbers refer to 2790 cm^−1^ (laser 1), 2926 cm^−1^ (laser 2) and 3350 cm^−1^ (laser 3). The data are mapped and aligned to each other. A region of interest (ROI) is defined for better visibility. Each image (RIO MIR and ROI FT-IR) consists of the mentioned wavenumbers. The similarity of the corresponding data sets is ~ 75%. Differences between the images are predominantly due to intensity differences.

**Figure 5:**
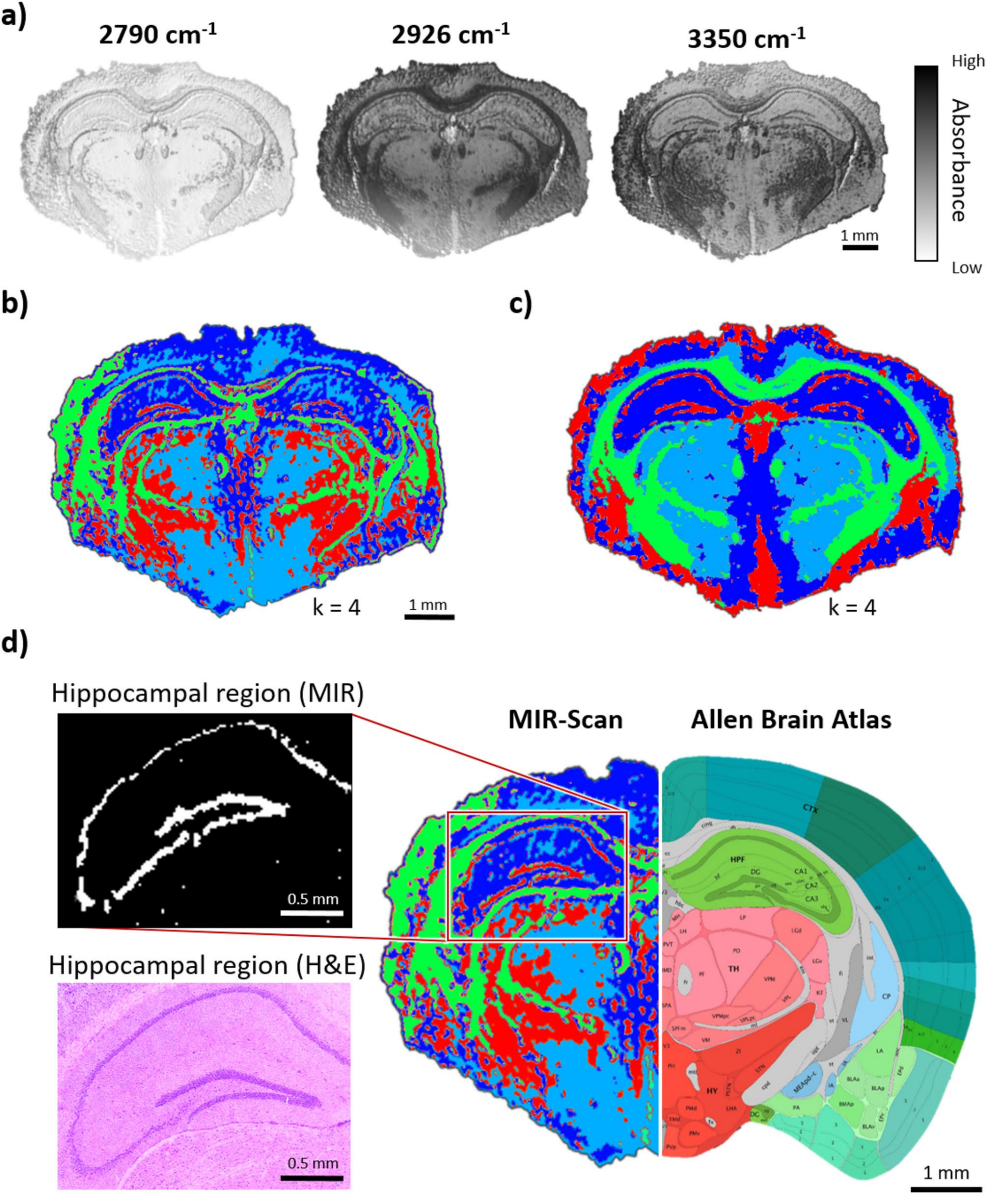
Comparison of measurement results for brain of mouse type C57BL/6 based on detection of hippocampal region by MIR scanner and FT-IR imager. Clustering via k-means is performed with k = 4 in each case. (**a**) Three wavenumbers measured by the MIR scanner. (**b**) k-means clustering for scan result of the MIR scanner. (**c**) k-means clustering for scan result of the FT-IR imager. (**d**) H&E staining of the hippocampal region in comparison of k-means clustering (MIR scanner) in relation to the Allen Brain Atlas for mice brain (2015 Allen Institute for Brain Science. Allen Brain Atlas API. Available from: brain-map.org/api/index.html)^54,55^. The hippocampal region is distinctly segmented as a coherent structure.

The spectral data from the MIR scanner and the FT-IR imager can be compared based on a similarity calculation. The similarity calculation indicates that the selected wavenumbers are comparable between the two measurement techniques. Therefore, the similarity calculation (Fig. 4) is based on the selected wavenumbers 2790 cm^−1^ (laser 1), 2926 cm^−1^ (laser 2) and 3350 cm^−1^ (laser 3) and are applied on the measurement results from Fig. 5. The data of both measurement techniques are mapped to each other. Subsequently, the individual data sets are compared via structural similarity index^46^. The index is composed of the calculation of the mean squared error (MSE)^47^. The similarity between the data sets is ~ 75%. The labelled region of interest shows that the structural variation between the measurement images is minor (Fig. 4). In contrast, the intensity difference between the measuring signals is significantly different and affects the similarity index.

The images produced by the MIR scanner (Fig. 5a) show different measurement images for different wavenumbers. Each scan result includes 250k measurement points, acquired within ~ 3 s. Grey matter (cortex and CA3 region) and white (corpus collosum) matter are visible for all wavenumbers. White matter has an absorption maximum at wavenumber 2926 cm^−1^ in the functional group region^48^. This is due to the increased CH_2_ or CH_3_ vibrations of white matter compared to grey matter^49^. The vibration of the NH-band, especially the vibration band of secondary amines near to wavenumber 3350 cm^−1^, is detected^50–52^. Significant differences between measurement results for wavenumber 2926 cm^−1^ and 3350 cm^−1^ are evident for some structures. Especially the hippocampus, due to its molecular composition, shows distinct differences in the vibrational properties of lipids and amines^51–53^. This is apparent in the measurement results too. For reference wavenumber 2790 cm^−1^, the hippocampal area is detected on a low absorbance level and thus represents an approximately ideal reference to this brain region. Despite the limited spectral information by the MIR scanner, clustering can be performed using a k-means algorithm. In this case, the focus is to achieve similar clustering results for MIR and FT-IR imaging. For both measurement methods, structures in the mouse brain are clustered (Fig. 5b,c). The direct comparison between the two measurement results proves that the essential structures such as the grey and white matter are clustered in a comparable way.

For clustering spectral data in this case, the MIR scan data consists only of three wavenumbers per pixel. In comparison, the spectral FT-IR dataset comprises over hundred wavenumbers each pixel. Due to the larger amount of information for the FT-IR measurement results, the clustering produces smoother structures and more homogeneous areas. In addition, some areas of the brain are more distinctly visible. This is due to the significant part of the spectral fingerprint area being included in the clustering of FI-IR images. However, for the measurement results of the MIR scanner, distinct structures are clustered, such as the white matter, the grey matter and the entire hippocampal region. Parts of thalamus and hypothalamus are rudimentary clustered too. The segmentation of the hippocampus is done at k-value k = 4. For measurement results via FT-IR imaging, the clustering and segmentation is done for k = 4 as well. But the mentioned k-value is related to clustering and segmentation on the specimen and represented by a certain colour in the resulting figures. Some cluster occurred on the background surface, so they are rejected. In comparison to the Allen Brain Mouse Atlas^54,55^, the region of the hippocampus is particularly well clustered for MIR scans (Fig. 5d). The resulting segmentation shows the hippocampus as an approximately coherent structure. The associated H&E staining of the sample confirms the measurement results of the MIR scanner regarding the hippocampal region.

### Examination of spontaneously occurring primary hepatocellular carcinoma

**Figure 6:**
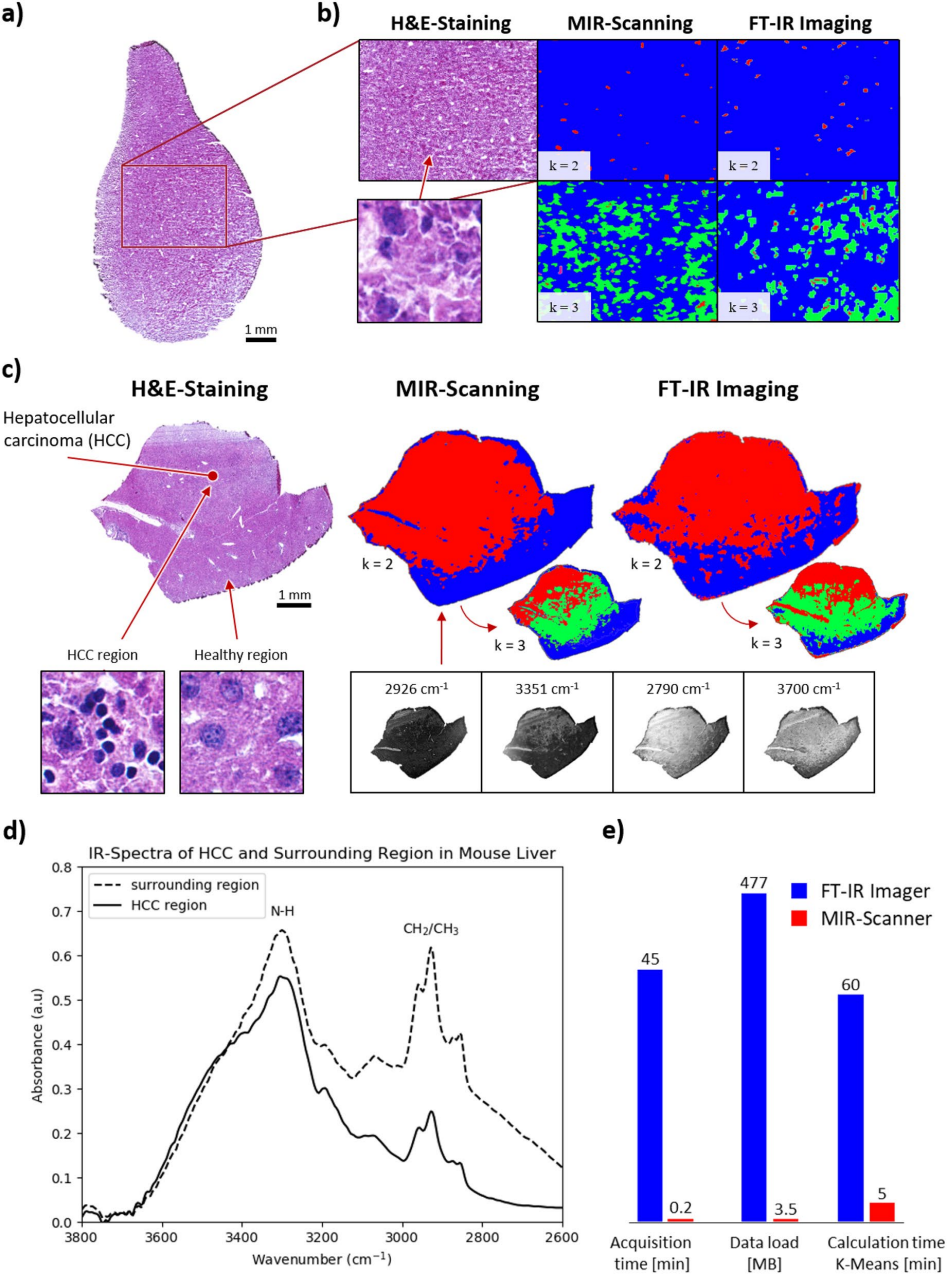
Scanning results for detection of histomorphologically healthy liver tissue and liver tumour (hepatocellular carcinoma). (**a**) H&E-stained section of healthy tissue of a mouse liver from a mouse type C57BL/6. (**b**) Cropped H&E section with k-means clustered comparison of MIR scanning and FT-IR imaging for k = 2 and k = 3. (**c**) H&E-stained HCC liver of mouse type BALB/c-Abcb4^−/−^ and k-means clustered scanning results for MIR scanning and FT-IR imaging (k = 2 and k = 3). (**d**) IR spectrum of tumourous tissue and surrounding area. (**e**) Comparison of the scanning methods with regard to scanning time, data load and calculation of k-means.

Based on successful segmentation of defined and specific structures in mouse brain, the next validation step for the MIR scanner is the investigation of not distinctly defined tissue structures. The examination of a spontaneous primary hepatocellular carcinoma (HCC) in mouse liver therefor offers suitable conditions since these usually show inhomogeneous tissue structures^56^. At the initiation of this investigation, it is necessary to ensure that the scanning systems used provide equivalent detection of healthy mouse livers (H&E staining, Fig. 6a) and ensure the resulting clustering provides comparable results by k-means. The H&E-stained section from Fig. 6a shows the location of the measurement performed in Fig. 6b on the sample. In addition, an magnified section (Fig. 6b) shows the morphological structure of healthy liver. This can be compared with the morphological structure (surrounding structure) from Fig. 6c.

This experiment is performed by using all four wavenumbers from the MIR scanner. The approximately homogeneous structure of a healthy mouse liver is detected with comparable parameters for both measurement systems, the MIR scanner and the FT-IR imager. The MIR scanner dataset for each laser consists of 375k measurement points, acquired at ~ 4.5 s. In Fig. 6b, the clustered scan results are shown next to the adjacent section stained by H&E. In this case, the entire tissue section is spectrally acquired and clustered for both measurement methods. Subsequently, the clustered measurement data is mapped on the H&E image. The image section is aligned and cropped identically for both data sets. Basically, the k-means algorithm has a high variance in reproducibility, thus the measurement results between MIR and FT-IR scan are not identical at all. For this reason, a pixel-by-pixel comparison is not valid. Nevertheless, the two methods show a very similar pattern for clustering k = 2 within healthy liver. For k = 3, similar segmentation results are obtained as well, but they differ from each other in size and location. This emphasizes the variance of the algorithm by using a different number of spectral data, but demonstrates that for low spatial scan resolutions and thus lower structural information, segmentation of approximately homogeneous tissue is challenging.

The examination of HCC is performed on a sample from mouse type BALB/c-Abcb4^−/−^^57,58^. By visual assessment of the diseased liver a tumour nodule of about 3 mm at the left lobe could be observed. The sample is chosen to include a transition from the nodule to surrounding tissue. This way, the detection of tumour margins of an infiltrated tumour is topographically feasible and provides direct comparison between tumour nodule and surrounding regions. The corresponding measurement results (250k measurement points each laser, ~ 3 s acquisition time each wavenumber; Fig. 6c) of the MIR scanner show a precise differentiation between the healthy and diseased tissue for clustering with k = 2. Higher k-values segment a transition area between healthy and diseased tissue. In comparison, measurement results for the FT-IR imager exhibit the same margins. However, the detection and clustering of tumour margins are slightly different for the two scanning methods. This effect might occur due to the different drying of blood within the liver. Blood vessels in the liver change their spectral properties in the N–H vibration band with increasing drying time^59^. The time between FT-IR and MIR measurement is about 2 h. Thus, the measurement result for this organ also changes slightly. In addition, the heterogeneous margin in both MIR images represents the infiltrating property of the tumour. Spectral analysis of the tumour nodule in relation to the surrounding area shows that these two regions are fundamentally different from each another (Fig. 6d). In this spectral range, it is predominantly the proportion of long-chain C-H molecules that indicates tissue changes within the liver. Based on cluster k = 3 (Fig. 6c), it can be concluded that a substructure within the HCC is detected. A more detailed investigation of the substructure is part of a further study. Therefore, it will not be discussed here. It should merely be pointed out that the MIR scanner can potentially detect such a structure comparable to the FT-IR imager and the current settings.

Both scanning methods (FT-IR and MIR) produced equivalent measurement results, although they are based on different spectral amount of information. Figure 6e summarises the main differences between FT-IR imager and MIR scanner. The comparison is based on scanning a square centimetre with spatial resolution of 25 µm for the FT-IR imager and 20 µm for the MIR scanner. The previous measurement images and measurement data are also generated by these parameters. In this case, the MIR scanner is scanning faster than the FT-IR imager by factor 225. The data load is reduced by factor 136 and the calculation time for data pre-processing and clustering is reduced at the MIR scanner by factor 10. The comparison is based on an established workflow^6^ and is intended to show the optimization of the workflow by the MIR scanner.

## Discussion

With the novel MIR scanner, it was demonstrated that the segmentation of selected tissue structures in mouse brain is possible by using selected wavenumbers in the low mid-infrared range. Here, the focus is on detection of lipid structures and structures associated with amide A. This results in the detection of the characteristic structure of the hippocampus. White and grey matter were detected with the MIR scanner as well. The measurement results of the MIR scanner are directly comparable to the results of FT-IR imaging in combination with k-means clustering. The MIR scanner provides just three or four spectral information per pixel, depending on the use case. In contrast, the pixels for segmentation with the FT-IR imager consist of more than a hundred spectral information sources, including the fingerprint area. Thus, some tissue structures are clustered more precise. Nevertheless, the MIR scanner is capable of providing comparable final results for specific applications based on reduced information content. The detection of naturally grown tumour in mouse liver (hepatocellular carcinoma) is also successfully performed by referencing with the FT-IR imager. However, the MIR scanning method has the major disadvantage that it can only detect selected fixed wavenumbers, which are not sufficient for every application. Despite this, we are able to accelerate the scanning process by a factor of 225 compared to FT-IR imaging. This reduces the data load by a factor of > 136 for the MIR scanner in comparison with an established workflow^6^.

Depending on the application, the laser modules have to be adapted or supplemented by FT-IR imaging. Nevertheless, considering this issue, a first fast analysis of a thin tissue section is achievable. As we have shown, the use of the MIR scanner is much faster than conventional reference methods^6,10,13,14^ for the rapid analysis of frozen sections. In this context, the presented MIR method does not replace H&E staining or even histological structural assessment, especially in the clinical field, by a pathologist. It can, though, be used as a supporting tool in the future.

Based on the measurement results presented here, the examination of further tissue sections (with tumor) of other organs such as spleen, kidney or heart is essential. In this context, the reduction of spatial scan resolution is a necessary requirement to enable the visualization of smaller structures and to perform further analyses. With a lower spatial resolution, an investigation of neurons within mouse brains would be feasible. There is potential for the detection of various plaques in the hippocampus as part of the investigation of Alzheimer's disease. In addition, based on the knowledge of automated guided MALDI (Matrix-Assisted Laser Desorption/Ionization) examinations^6^, the link between MIR scanner and MALDI MSI (Mass Spectrometry Imaging) is made available. The rapid pre-diagnosis by the MIR scanner enables to select predefined regions of interest within the tissue for MALDI MSI analysis that reduce acquisition time for high-resolution data with low spatial resolution. Furthermore, it avoids unused data that occur with measurement ranges that are not relevant. Thus, a more efficient use of MALDI MSI can be increased.

Considering the wavenumbers used, the MIR scanner provides significant added value for clinical applications as well. After validating the measurement setup on human tissue samples, the MIR scanner can support clinical frozen section analysis. With sufficient knowledge of the sample composition of lipids and proteins, the pathologist can also use the MIR scanner as a pre-processing tool for further investigations. Thus, for larger samples, a segmentation of relevant tissue regions or tumour areas could be performed, which the pathologist confirms with histopathological knowledge. For this topic, further studies with the MIR scanner need to be initiated to prove its feasibility.

Furthermore, the reliability of the measurement data has to be developed. In particular, the reproducibility of the clustered measurement results via k-means needs to be ensured. The dynamic determination of the individual clusters during the calculation of k-means is problematic, which considerably limits the reproducibility. However, even from this point of view, we were still able to deliver comparable and usable results for the frozen section analysis.

Taken together, this study showed a novel none-destructive measurement system for fast structure and tumour detection. We proved our measuring setup by referencing our results with a commercially available FT-IR imaging system. Due to this we demonstrated a new rapid analytical opportunity for intraoperative frozen tissue section analysis or rapid pre-analysis for MALDI MSI.

## Methods

### Optical characterisation and settings of the MIR scanner

Based on the measurement of absorbance differences depending on molecule vibrations, the MIR scanner provided measurement points representing the vibration intensity of the local molecules of the sample. Thus, a measuring point represented an intensity value which was due to the local concentration related absorption behaviour of the sample was examined. The absorption referred to the background of the sample, especially to the absorption characteristic of the microscope slide. Ideally, the slide was coated with gold or silver in order to be able to make a comparable statement of the absorption via FT-IR or DRIFTS (Diffuse Reflectance Infrared Fourier Transform Spectroscopy). In our experiments, silver-coated slides (Kevley Technologies, Ohio) were used because gold-coated slides did not provide a beneficial difference.

The entire optical setup of the MIR scanner was designed for the measurement of functional groups. Thus, the design of the optical components was also focused to this spectral range. The spectral range to be detected was between 2000 cm^−1^ and 4000 cm^−1^. The IR detector was configured for this spectral range and had its detection maximum at this specific spectral region. The single element sensor had a sensor area of 1 × 1 mm^2^ and was four-stage pre-amplified. A sapphire window with anti-reflective coating was located in front of the sensor chip. Thus, the incident collimated measuring signal was projected optimally onto the sensor chip. In addition, all lenses (CaF_2_ and Black Diamond) of the MIR scanner were coated with an anti-reflective coating. The IR detector and the lens coating improved the optical performance of the MIR scanner in this spectral range, but limited it at the same time. To remove the spectral limitation caused by lenses, the coating of lenses in combination with the lens material had to be modified, depending on the application^60–62^. Optical components from the align mirror unit consisted of a unit called ELEFHANT Precession (Novanta Europe GmbH, Germany). The including mirrors were gold-coated to improve the reflective properties in the middle infrared spectral range. The focus unit consisted of a combination of several lenses (CaF_2_, anti-reflective coated for the spectral range between 4000 cm^−1^ and 2000 cm^−1^). The lens system was able to automatically adjust the focus point. Therefore, a focus shift due to the wavelength were adjusted. The lens system had a numerical aperture of 0.12 and a magnification of 1.25.

The MIR scanner had four wavenumbers that were used for measurements. The individual wavenumbers were represented by Distributed Feedback (DFB) lasers (nanoplus Nanosystems and Technologies GmbH, Germany) that illuminated the sample depending on the measurement and necessity^37,38^. A lens system was used to focus the laser light onto the sample.

Measurements with the MIR-Scanner were performed placing the sample on the 2D translation stage. The translation stage moved automatically into the predefined scanning field of the MIR scanner. The scanning velocity of the align mirror was set to 5.3 m/s with a spatial resolution of 20 µm. For a laser line of 1 cm length, this resulted in 5k measurement points (including oversampling with factor 10). The mirror velocity predominantly defined the sample velocity of the data and the velocity of the translation stage. For 5k measurement points per measurement line (in this case a scanning field 1 × 1 cm^2^), a sample rate of ~ 2.7 MS/s and a translation stage speed of ~ 3.3 mm/s resulted. For each measuring line, an offset of 0.5 cm was included at the beginning and end of the line, to ensure that the scanning speed was constant in the measuring range. The parameters referred to a corrected image field and a working distance of ~ 2 cm. Without calibration of the image field, image distortions occurred because the mirror deflection did not match the target pathway on the sample. Using different wavenumbers, the lasers were initiated sequentially. A background correction was performed via a reference measurement for each laser. The reference measurement was performed on the substrate the specimen was located on, in this case on a Mirr-IR slide (Kevley Technologies, Ohio). The background measurement was then calculated with the measurement signal of the respective laser. In addition, the measurement images were pre-processed and calculated by the described workflow.

### FT-IR imaging

The FT-IR measurement results listed in this work were acquired with the Perkin Elmer Spotlight 400 FT-IR imager^6^ (Perkin Elmer Inc., USA). With the FT-IR imager, the spectrum from 4000 to 750 cm^−1^ was measured for each experiment. The spectral resolution was set to 8 cm^−1^. The measurements were made in reflexion mode, as this is closest to the scanning method of the MIR scanner. Each measurement point was accumulated twice to reduce background noise. The scanning speed was set to 2.2 cm/s. Before measurement, the detector of the FT-IR imager was cooled with liquid nitrogen.

### Pre-processing und k-means

The measurement results were pre-processed to prepare the data from the MIR scanner and the FT-IR imager for the k-means algorithm. The results of the MIR scanner were aligned to each other so that the position of each pixel is the same for all images. A threshold adjustment was applied to amplify particularly weak signals. Subsequently, the measurement data was denoised. For segmentation and clustering of the individual tissue structures, k-means clustering was used. The first two k-values basically could not be used for the evaluation, because these were related to the background and the associated segmentation between slide and tissue. So, k-values related to the background were rejected.

The spectral data sets of the FT-IR imager were denoised and normalized afterwards. A smoothing and normalization of the spectra was used to purify the measurement data. Subsequently, spectral ranges were defined that were to be considered in the clustering via k-means. The spectral range was set to the functional groups and fingerprint region.

Pre-processing for MIR scanning data was performed by using python (https://www.python.org/). MATLAB (The MathWorks Inc., USA) was used for FT-IR data pre-processing, general k-means clustering and segmentation^6^.

### Sample preparation and H&E staining

The tissue samples were cut in 10 µm sections using a CM1950 cryostat (Leica Biosystems Nussloch GmbH, Germany). Before and after cutting the tissue sample, the organ was stored at − 80 °C in a freezer. The frozen tissue samples were placed and thaw-mounted on a Mirr-IR slide (Kevley Technologies, Ohio). Samples used for H&E-staining were thaw-mounted on a SuperFrost Plus slide (Thermo Fisher Scientific Inc., USA). Finally, the samples for MIR and FT-IR measurements were dried using a desiccator at low pressure for 10 min.

H&E staining was performed by removing hematoxylin after ~ 2 min, followed by washing in tap water for ~ 3 min, one dip in distilled water and ~ 1 min acidic alcohol. Then, the sample was dipped in distilled water three times and put into blueing solution for ~ 2 min. In addition, blueing solution was removed by three drips in distilled water. Eosin (0.5% aqueous) was removed after ~ 2 min by ~ 1 min distilled water. Washing the samples was performed by 80% and 90% ethanol for ~ 2 min each and two times in 100% ethanol for ~ 1 min each. The samples were final cleaned by ~ 2 min xylene. After H&E staining the samples were mounted with Eukitt (Sigma Aldrich GmbH, Germany) and protected with cover glass.

### Animal specimens

Animal studies conducted at the Universitätsklinikum Mannheim (UMM) were supervised by institutional animal protection officials in accordance with the National Institute of Health guidelines Guide for the Care and Use of Laboratory Animals. The animal experiments were approved by governmental authorities (Regierungspräsidium Karlsruhe, Germany, approval number: G172/15 for mouse strain BALB/c-abcb4^−/−^ and I-20/08 for mouse strain C57BL/6). The BALB/c-abcb4^−/−^ mouse strain^58^ used to investigate primary infiltrating liver tumorous tissue carries a homozygous deletion of the gene encoding the drug transporter ABCB4. This strain develops spontaneous hepatic fibroses homologous to the phenotype of sclerosing cholangitis. Thus, hepatocellular carcinoma (HCC), which is a primary malignancy originating in the liver, is often spontaneously developed by the 9–12th month of their life span. Liver harvesting was conducted at a 548 days old male mouse of the described strain. A part of the left lobe of the liver was immediately snap-frozen by the use of liquid nitrogen.

## References

[1] Asem M, Abudalu LE (2019). Accuracy of frozen-section diagnosis of brain tumors: An 11-year experience from a tertiary care center. Turk. Neurosurg..

[2] Tepe NB (2019). Is intraoperative frozen examination sufficiently reliable for ovarian tumors: 11 years experience at a single center. Eur. J. Gynaecol. Oncol..

[3] Sharifabadi AH (2016). Intraoperative consultation of central nervous system lesions. Frozen section, cytology or both?. Pathol. Res. Pract..

[4] Pandey S, Bhamra S, Singh A (2020). Accuracy of intraoperative frozen section in assessing margins in oral cancers: A tertiary care hospital based study. IJPO.

[5] Tuck M (2020). Multimodal imaging based on vibrational spectroscopies and mass spectrometry imaging applied to biological tissue: A multiscale and multiomics review. Anal. Chem..

[6] Rabe J-H (2018). Fourier transform infrared microscopy enables guidance of automated mass spectrometry imaging to predefined tissue morphologies. Sci. Rep..

[7] Mehrotra R, Tyagi G, Jangir DK, Dawar R, Gupta N (2010). Analysis of ovarian tumor pathology by Fourier transform infrared spectroscopy. J. Ovarian Res..

[8] Wrobel TP, Mateuszuk L, Chlopicki S, Malek K, Baranska M (2011). Imaging of lipids in atherosclerotic lesion in aorta from ApoE/LDLR^−^^/^^−^ mice by FT-IR spectroscopy and hierarchical cluster analysis. Analyst.

[9] Kröger-Lui N (2015). Rapid identification of goblet cells in unstained colon thin sections by means of quantum cascade laser-based infrared microspectroscopy. Analyst.

[10] Kilgus J (2018). Diffraction limited mid-infrared reflectance microspectroscopy with a supercontinuum laser. Opt. Express.

[11] Guo B (2004). Laser-based mid-infrared reflectance imaging of biological tissues. Opt. Express.

[12] Kuepper C (2018). Quantum cascade laser-based infrared microscopy for label-free and automated cancer classification in tissue sections. Sci. Rep..

[13] Krafft C (2017). Label-free molecular imaging of biological cells and tissues by linear and nonlinear raman spectroscopic approaches. Angew. Chem. (International Ed. in English).

[14] Krafft C (2009). A comparative Raman and CARS imaging study of colon tissue. J. Biophoton..

[15] Isensee K, Kröger-Lui N, Petrich W (2018). Biomedical applications of mid-infrared quantum cascade lasers—A review. Analyst.

[16] Mittal S (2018). Simultaneous cancer and tumor microenvironment subtyping using confocal infrared microscopy for all-digital molecular histopathology. Proc. Natl. Acad. Sci. U.S.A..

[17] Krishnamurthy S (2018). Ex vivo confocal fluorescence microscopy for rapid evaluation of tissues in surgical pathology practice. Arch. Pathol. Lab. Med..

[18] Hollon TC (2018). Rapid intraoperative diagnosis of pediatric brain tumors using stimulated Raman histology. Cancer Res..

[19] Hill AH, Manifold B, Fu D (2020). Tissue imaging depth limit of stimulated Raman scattering microscopy. Biomed. Opt. Express.

[20] Bae K, Zheng W, Huang Z (2020). Spatial light-modulated stimulated Raman scattering (SLM-SRS) microscopy for rapid multiplexed vibrational imaging. Theranostics.

[21] Conway RM, Themel S, Holbach LM (2004). Surgery for primary basal cell carcinoma including the eyelid margins with intraoperative frozen section control: Comparative interventional study with a minimum clinical follow up of 5 years. Br. J. Ophthalmol..

[22] Shah JS (2019). Accuracy of intraoperative frozen section diagnosis of borderline ovarian tumors by hospital type. J. Minim. Invasive Gynecol..

[23] Ivkovic-Kapicl T (2020). Intraoperative imprint cytology of sentinel lymph nodes in breast cancer patients: Comparation with frozen section. VSP.

[24] Heller B, Peters S (2011). Assessment of liver transplant donor biopsies for steatosis using frozen section: Accuracy and possible impact on transplantation. J. Clin. Med. Res..

[25] Lechago J (2005). Frozen section examination of liver, gallbladder, and pancreas. Arch. Pathol. Lab. Med..

[26] Miedema JR, Hunt HV (2010). Practical issues for frozen section diagnosis in gastrointestinal and liver diseases. J. Gastrointest. Liver Dis..

[27] Mair S, Lash R, Suskin D, Mendelsohn G (1991). Intraoperative surgical specimen evaluation: Frozen section analysis, cytologic examination, or both?. Am. J. Clin. Pathol..

[28] van den Bent MJ (2010). Interobserver variation of the histopathological diagnosis in clinical trials on glioma: A clinician's perspective. Acta Neuropathol..

[29] Gilles FH (2008). Pathologist interobserver variability of histologic features in childhood brain tumors: Results from the CCG-945 study. Pediatr. Dev. Pathol..

[30] Sondermann W (2016). Initial misdiagnosis of melanoma located on the foot is associated with poorer prognosis. Medicine.

[31] Zhu X, Bledsoe JR (2020). Frozen section diagnosis of gastrointestinal poorly cohesive and signet-ring cell adenocarcinoma: Useful morphologic features to avoid misdiagnosis. Virchows Arch..

[32] Huang Z (2018). Diagnostic accuracy of frozen section analysis of borderline ovarian tumors: A meta-analysis with emphasis on misdiagnosis factors. J. Cancer.

[33] Yoshida H (2020). Diagnostic discordance in intraoperative frozen section diagnosis of ovarian tumors: A literature review and analysis of 871 cases treated at a Japanese cancer center. Int. J. Surg. Pathol..

[34] Hatwig, J., Minnerup, P., Zaeh, M. F. & Reinhart, G. An automated path planning system for a robot with a laser scanner for remote laser cutting and welding. In *2012 IEEE International Conference on Mechatronics and Automation* (IEEE Sunday, August 5, 2012–Wednesday, August 8, 2012), 1323–1328.

[35] Pieczona, S. J., Zollitsch, S. & Zaeh, M. F. *2017 IEEE International Conference on Advanced Intelligent Mechatronics *(*AIM*),* 3–7 July 2017* (IEEE, 2017).

[36] Kachelriess M, Knaup M, Penssel C, Kalender WA (2006). Flying focal spot (FFS) in cone-beam CT. IEEE Trans. Nucl. Sci..

[37] Kümmel T (2019). Contrast enhancement of surface layers with fast middle-infrared scanning. Heliyon.

[38] Beyerer J, León FP, Längle T (2019). OCM 2019—Optical characterization of materials: Conference proceedings. Opt. Charact. Mater..

[39] Lozano M (2017). Mid-infrared spectroscopy (MIR) for simultaneous determination of fat and protein content in meat of several animal species. Food Anal. Methods.

[40] Duygu D (2012). Fourier transform infrared (FTIR) spectroscopy for identification of *Chlorella**vulgaris* Beijerinck 1890 and *Scenedesmus**obliquus* (Turpin) Kützing 1833. Afr. J. Biotechnol..

[41] Szentirmai V (2020). Reagent-free total protein quantification of intact extracellular vesicles by attenuated total reflection Fourier transform infrared (ATR-FTIR) spectroscopy. Anal. Bioanal. Chem..

[42] Grasmair, M. Locally adaptive total variation regularization. *Scale Space and Variational Methods in Computer Vision*, 331–342. 10.1007/978-3-642-02256-2_28 (2009).

[43] Likas A, Vlassis N, Verbeek Jakob J (2003). The global k-means clustering algorithm. Pattern Recognit..

[44] Galea I, Bechmann I, Perry VH (2007). What is immune privilege (not)?. Trends Immunol..

[45] Swanson LW, Hahn JD (2020). A qualitative solution with quantitative potential for the mouse hippocampal cortex flatmap problem. Proc. Natl. Acad. Sci. U.S.A..

[46] Wang, Z., Bolvik, A. C., Sheikh, H. R. & Simoncelli, E. P. Image quality assessment: From error visibility to structural similarity. *IEEE Transactions of Image Processing,* 600–612. 10.1109/TIP.2003.819861 (2004). 10.1109/tip.2003.819861 15376593

[47] Wang Z, Bovik AC (2009). Mean squared error: Love it or leave it? A new look at signal fidelity measures. IEEE Signal Process. Mag..

[48] Kidder LH (1999). Infrared spectroscopic imaging of the biochemical modifications induced in the cerebellum of the Niemann–Pick type C mouse. J. Biomed. Opt..

[49] Sanchez-Molina P (2020). From mouse to human: Comparative analysis between grey and white matter by synchrotron-Fourier transformed infrared microspectroscopy. Biomolecules.

[50] Dovbeshko GI, Gridina NY, Kruglova EB, Pashchuk OP (2000). FTIR spectroscopy studies of nucleic acid damage. Talanta.

[51] Petibois C, Déléris G (2006). Chemical mapping of tumor progression by FT-IR imaging: Towards molecular histopathology. Trends Biotechnol..

[52] Palombo F (2018). Detection of Aβ plaque-associated astrogliosis in Alzheimer's disease brain by spectroscopic imaging and immunohistochemistry. Analyst.

[53] Hackett MJ (2019). Multimodal imaging analyses of brain hippocampal formation reveal reduced cu and lipid content and increased lactate content in non-insulin-dependent diabetic mice. ACS Chem. Neurosci..

[54] Jones AR, Overly CC, Sunkin SM (2009). The Allen Brain Atlas: 5 years and beyond. Nat. Rev. Neurosci..

[55] Lein ES (2007). Genome-wide atlas of gene expression in the adult mouse brain. Nature.

[56] Calderaro J (2017). Histological subtypes of hepatocellular carcinoma are related to gene mutations and molecular tumour classification. J. Hepatol..

[57] Henkel C (2006). Changes of the hepatic proteome in murine models for toxically induced fibrogenesis and sclerosing cholangitis. Proteomics.

[58] Katzenellenbogen M (2007). Molecular mechanisms of liver carcinogenesis in the mdr2-knockout mice. Mol. Cancer Res. MCR.

[59] Zou Y (2016). Whole blood and semen identification using mid-infrared and Raman spectrum analysis for forensic applications. Anal. Methods.

[60] Schnell M (2020). All-digital histopathology by infrared-optical hybrid microscopy. Proc. Natl. Acad. Sci. U.S.A..

[61] Phal Y, Yeh KL, Bhargava R (2020). Polarimetric infrared spectroscopic imaging using quantum cascade lasers. Adv. Chem. Microsc. Life Sci. Transl. Med..

[62] Yeh K, Lee D, Bhargava R (2019). Multicolor discrete frequency infrared spectroscopic imaging. Anal. Chem..

